# Development and Implementation of a Peer-Teaching Curriculum for Student Assistants

**DOI:** 10.1007/s40670-024-02050-8

**Published:** 2024-04-30

**Authors:** Meike Dirmeier, Kristina Schick, Kathleen Lindemann, Larissa Bethenod, Marjo Wijnen-Meijer

**Affiliations:** 1 Technical University of Munich, TUM School of Medicine & Health, Student Office, Munich, Germany; 2 Technical University of Munich, TUM School of Medicine & Health, Department Clinical Medicine, TUM Medical Education Center, Munich, Germany, Munich, Germany; 3 Institute of Medical Education, Medical Faculty and University Hospital Carl Gustav Carus, TUD Dresden University of Technology, Dresden, Germany

**Keywords:** Peer-teaching program, Medical education, Student assistants, Teaching methodology, Curriculum development

## Abstract

We have developed a peer-teaching program for student assistants involved in medical education. The offer comprises (1) an inventory of potentially relevant courses offered by other institutions at our university and (2) our own peer-teaching curriculum on pedagogy and teaching methodology. We describe a pilot scheme to implement the curriculum.

Since almost all physicians have teaching responsibilities, it is important to prepare students in medical school for this future role [1]. Research shows that didactic training and gaining early teaching experiences have positive effects on the development of students’ communication skills and learning, and also the extent to which they later fulfil an active role in (medical) education [2]. Student peer teaching fosters a pleasant learning environment, reduced exam-related stress, and is a useful adjunct to faculty teaching [3]. Peer teachers can focus on smaller tasks and thus reduce lecturers’ workloads, especially for simpler skills such as blood sampling and ultrasound. Many medical schools therefore offer teacher qualification programs as part of the (compulsory or elective) undergraduate medical curriculum [1, 2].

As neither compulsory nor elective curricular embedding is currently possible at the medical school of the Technical University of Munich (TUM), we adopted a different approach: We developed a peer-teaching program for student assistants in medical education. This approach has three objectives: (i) to enhance the teaching competences of our student assistants, (ii) to strengthen commitment to the TUM Medical Education Center, and (iii) to foster students’ motivation for their (future) teaching role as clinical physicians.

TUM Medical Education Center employs about 70 student assistants per semester from study years 2 to 6 and from different disciplines related to healthcare (mainly medicine). For our pilot scheme, we selected student assistants based on a non-standardized recruitment interview. The student assistants were deployed in different fields of work, depending on their interests, e.g., skills labs, medical education research, eScouts, and departmental support. Our program was specifically designed for student assistants in year 3 or later, since previous research findings recommend senior student assistants are more suitable due their more extensive experience of medical school [3]. The peer teachers’ tasks comprised developing teaching approaches, teaching, and/or advising, for example, peer-teachers of skills courses or case-based clinical reasoning courses, or so-called eScouts,^1^ who produce instructional videos and collaborate with teachers to develop and implement innovative digital teaching materials. However, other student assistants were also welcome to participate in our peer-teaching program.

Our training program aims to foster the specific educational competences of our student assistants, to introduce them to the specific teaching conditions at TUM Medical School (e.g., technical equipment), and encourage exchange between our student assistants.

To develop our teaching training program, we used a three-step approach: (1) a needs analysis to identify which skills and competences students require to successfully fulfil their assistant tasks; (2) an overview of suitable courses already offered by other institutions at our university, for example a literature search course at the university library; and (3) to cover additional needs, we developed our own training modules.

Regarding (1): For our needs analysis, we contacted student assistant supervisors via e-mail and asked them to list the tasks their student assistants have to perform. The analyses of their responses were counted and comparable answers were aggregated. The final list of topics included didactic principles, giving feedback, written and oral communication, handling IT equipment, and research skills (i.e., literature management, data management and analysis, using evaluation tools).

Regarding (2): Based on the results of the needs analysis, we identified courses that are already available to our student assistants to train the required skills and competences. This overview of courses already offered by TUM is published on an intranet page accessible to student assistants. At the beginning of each semester, the overview is updated, and the link is sent to the student assistants. Student assistants take part in the courses during working hours and are therefore paid for their participation in the course. A selection of these courses are shown in Fig. 1.

**Fig. 1:**
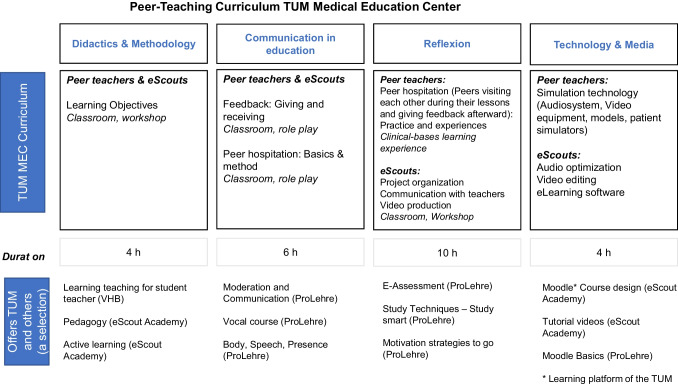
Overview peer-teaching curriculum TUM Medical Education Center

Regarding (3): To practice the competences that could not be covered by the courses already offered by other institutions, we developed our own training program with an inter-professional team consisting of psychologists, educational scientists, educationalists specialized in multimedia, and sociologists. The training program consists of four modules, where the contents of modules 1 and 2 are identical for all student assistants, while modules 3 and 4 provide task-specific training (Fig. 1). The modules were developed for a group size of 12–15 medical students and were to be completed within the first year of employment.

The students who participated in (parts of) the training program received a certificate. It is envisaged that in future this can be counted as part of the (clinical) teacher professionalization program.

In the pilot scheme with seven student assistants on the program, student assistants’ initial reactions were positive. They felt better prepared for their tasks and found the exchange with other student assistants valuable.

As a next step, we plan to conduct a follow-up study to evaluate the learning outcomes and students’ perspectives on the training programs to improve our approach.

## Footnotes

1 For further information about our eScout see also https://www.meditum.med.tum.de/de/content/escouts-f%C3%BCr-die-medizinische-lehre
